# The complete plastid genome and phylogenetic analysis of *Gracilaria edulis*

**DOI:** 10.1080/23802359.2019.1642161

**Published:** 2019-07-18

**Authors:** Tao Liu, Xianming Tang, Xuli Jia, Xiangyu Wu, Min Huang, Jun Zeng, Weizhou Chen

**Affiliations:** a Hainan Academy of Ocean and Fisheries Sciences, Haikou, People’s Republic of China;; b Hainan Provincial Key Laboratory of technology for tropical seawater aquaculture, Haikou, People’s Republic of China;; c Laboratory of Genetics and Breeding of Marine Organism, College of Marine Life Sciences, Ocean University of China, Qingdao, People’s Republic of China;; d Marine Biology Institute, Shantou University, Shantou, People’s Republic of China

**Keywords:** *Gracilaria edulis*, plastid genome, Gracilariaceae, phylogenetic analysis

## Abstract

*Gracilaria edulis*, a marine red macroalgae, is a rich source of sulfated polysaccharides, carbohydrate, vitamins, and minerals, and showed multiple bioactivities such as antibacterial, antitumour, and cholinesterase inhibitory activity. The plastid genome sequence of *G. edulis* is 179,410 bp. A total of 235 genes were determined, including 201 protein-encoding genes, 30 tRNA genes, 3 rRNA genes, 1 ribonuclease gene, and 1 intron inserted into the *trnM* gene. Phylogenetic analysis showed that *G. edulis* clustered together with *Gracilaria salicornia*, *Gracilaria tenuistipitata* var. *liui* and *Gracilaria chilensis.* The plastid genome analysis will help in the understanding of *Gracilaria* evolution.

*Gracilaria edulis* (S.G.Gmelin) P.C.Silva is a marine red alga belonging to the family Gracilariaceae (http://www.algaebase.org/). *Fucus edulis* S.G.Gmelin is the basionym of *G. edulis*. The species first came to attention because this previous edible alga contained a toxin causing fatal human poisoning (Yotsu-Yamashita et al. 2004). The following studies were about the physicochemical properties, nutritional composition, mineral and trace metals concentrations of *G. edulis* (Sakthivel and Pandima Devi 2015; Thodhal Yoganandham et al. 2018). In addition, Most of the research focused on its bioactivities such as antitumour activity (Patra and Muthuraman 2013; Priyadharshini et al. 2014; Sakthivel et al. 2016) and cholinesterase inhibitory activity which can be used in Alzheimer's disease treatment (Suganthy et al. 2010). Some studies explored its usage in withanolides production (Sivanandhan et al. 2013, 2015) and biodiesel production (Bharathiraja et al. 2016). However, no genomic studies on *G. edulis* have been reported.

In this study, we report the determination of the complete *G. edulis* plastid genome sequence by next-generation sequencing methods. The genomic DNA collected from one *G. edulis* individual in a population in south China (Yinggehai, Hainan Province, 18°30′36′′ N, 108°42′15′′ E) was sequenced. The specimen was stored at the Culture Collection of Seaweed at the Ocean University of China (sample accession number: 2017060064). Paired-end reads (150 bp) were sequenced by using Illumina HiSeq system (Illumina, San Diego, CA, USA), obtaining 27 Gb of sequence data. The tRNA genes were identified by using tRNAscan-SE Search Server (Schattner et al. 2005). Other plastid genomic regions were annotated from the *Gracilaria chilensis* (NC_029860) plastid genome by using Geneious R10 (Biomatters Ltd., Auckland, New Zealand).

The complete *G. edulis* plastid genome is a circular DNA molecule measuring 179,410 bp in length, and the overall G + C content of the complete plastid genome was 30.0% (GenBank accession number MN053318). The plastid genome contained 235 genes, including 201 protein-coding genes, 1 ribonuclease gene (*rnpB*), 3 rRNA genes, 30 tRNA genes, and 1 intron interrupting the *trnM* gene. The length of the coding region was 143,982 bp, corresponding to 80.3% of the total length. The plastid genome of *G. edulis* was compact, with 10 pairs of overlapping genes found with overlap lengths of 2–95 bp (*rps18*–*rpl33*, *atpF*–*atpD*, *trnR*–*chlI*, *carA*–*ycf53*, *psbD*–*psbC*, *ycf40*–*rps1*, *trnT*–*ilvB*, *rpl14*–*rps17*, *rps17*–*rpl29*, and *rpl23*–*rpl4*). The gene numbers and structures were largely similar among Gracilariaceae species published in the NCBI sequence database.

Phylogenetic analysis was conducted using MrBayes 3.1.2 software (Ronquist and Huelsenbeck 2003) based on 81 shared plastid protein sequences from 17 red algal plastid genomes and *Cyanidioschyzon merolae* (NC_004799) served as the outgroup. Concatenated alignments were generated and poorly aligned regions were removed by using the Gblocks server (Castresana 2000). All red algal taxa were clearly separated according to their original class (Figure 1). Florideophyceae species formed a large branch, in which The *Gracilaria* species formed a sub-branch including *G. edulis.* This analysis of complete plastid genome is conducive to understand the evolution of *Gracilaria*.

**Figure 1.**
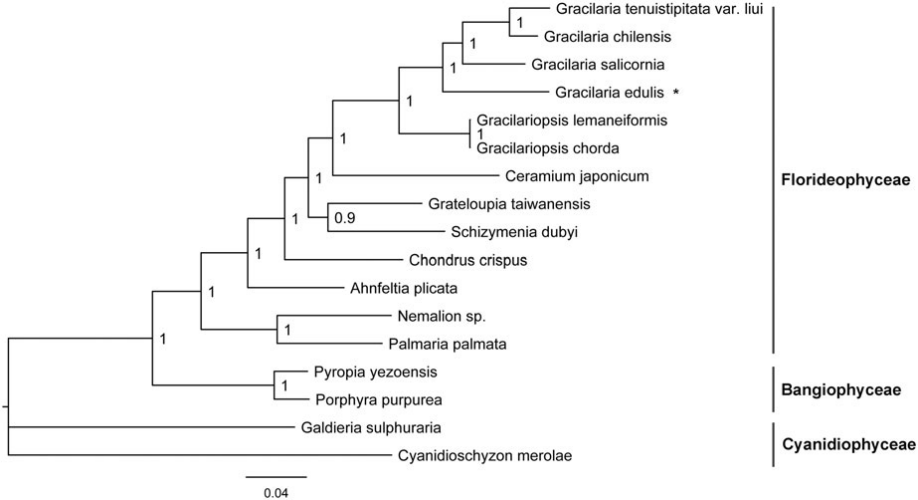
Phylogenetic tree (Bayesian method) based on the complete plastid genome sequence of red algae as shown below: *Gracilaria edulis* (MN053318), *Gracilaria salicornia* (NC_023785), *Gracilaria tenuistipitata* var. *liui* (AY673996), *Gracilaria chilensis* (NC_029860), *Gracilariopsis chorda* (NC_031149), *Gracilariopsis lemaneiformis* (KP330491), *Grateloupia taiwanensis* (KC894740), *Schizymenia dubyi* (NC_031169), *Chondrus crispus* (NC_020795), *Ceramium japonicum* (NC_031174), *Nemalion* sp. (LT622871), *Ahnfeltia plicata* (NC_031145), *Palmaria palmata* (NC_031147), *Pyropia yezoensis* (KC517072), *Porphyra purpurea* (U38804), *Galdieria sulphuraria* (KJ700459), and *Cyanidioschyzon merolae* (NC_004799). The asterisks after species names indicate newly determined plastid genomes.
